# Multiomics insights on the onset, progression, and metastatic evolution of breast cancer

**DOI:** 10.3389/fonc.2023.1292046

**Published:** 2023-12-19

**Authors:** Lucia Alvarez-Frutos, Daniel Barriuso, Mercedes Duran, Mar Infante, Guido Kroemer, Roberto Palacios-Ramirez, Laura Senovilla

**Affiliations:** ^1^ Laboratory of Cell Stress and Immunosurveillance, Unidad de Excelencia Instituto de Biomedicina y Genética Molecular (IBGM), Universidad de Valladolid – Centro Superior de Investigaciones Cientificas (CSIC), Valladolid, Spain; ^2^ Laboratory of Molecular Genetics of Hereditary Cancer, Unidad de Excelencia Instituto de Biomedicina y Genética Molecular (IBGM), Universidad de Valladolid – Centro Superior de Investigaciones Cientificas (CSIC), Valladolid, Spain; ^3^ Centre de Recherche des Cordeliers, Equipe labellisée par la Ligue contre le cancer, Université Paris Cité, Sorbonne Université, Inserm U1138, Institut Universitaire de France, Paris, France; ^4^ Metabolomics and Cell Biology Platforms, Institut Gustave Roussy, Villejuif, France; ^5^ Department of Biology, Institut du Cancer Paris CARPEM, Hôpital Européen Georges Pompidou, Paris, France

**Keywords:** breast cancer, early-stage, cancer progression, metastasis, omics, biomarkers

## Abstract

Breast cancer is the most common malignant neoplasm in women. Despite progress to date, 700,000 women worldwide died of this disease in 2020. Apparently, the prognostic markers currently used in the clinic are not sufficient to determine the most appropriate treatment. For this reason, great efforts have been made in recent years to identify new molecular biomarkers that will allow more precise and personalized therapeutic decisions in both primary and recurrent breast cancers. These molecular biomarkers include genetic and post-transcriptional alterations, changes in protein expression, as well as metabolic, immunological or microbial changes identified by multiple omics technologies (e.g., genomics, epigenomics, transcriptomics, proteomics, glycomics, metabolomics, lipidomics, immunomics and microbiomics). This review summarizes studies based on omics analysis that have identified new biomarkers for diagnosis, patient stratification, differentiation between stages of tumor development (initiation, progression, and metastasis/recurrence), and their relevance for treatment selection. Furthermore, this review highlights the importance of clinical trials based on multiomics studies and the need to advance in this direction in order to establish personalized therapies and prolong disease-free survival of these patients in the future.

## 1 Introduction

**Figure 1:**
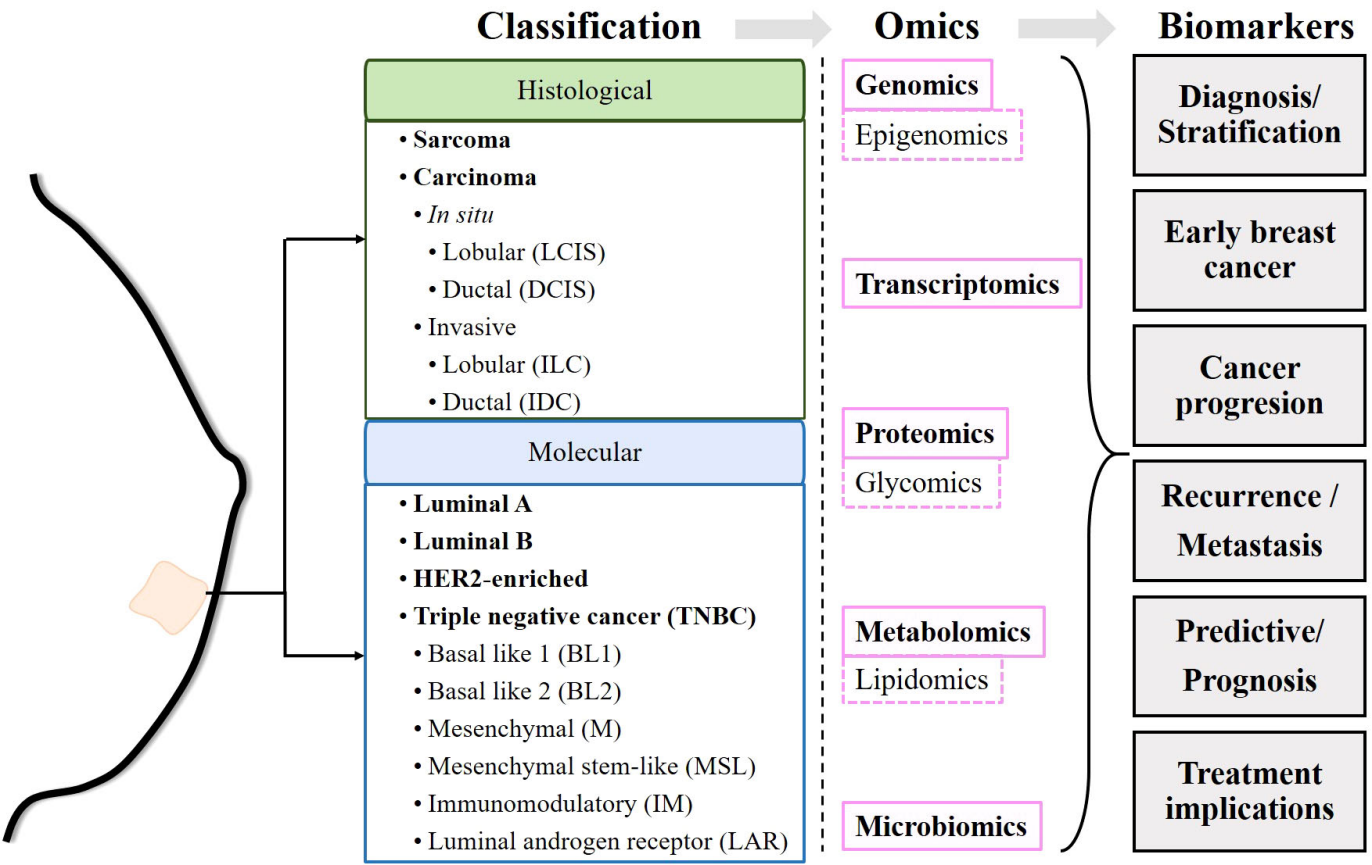
Schematic of the workflow performed in the review. Once the commonly accepted classifications of breast cancer have been described, the biomarkers identified by different omics techniques are described.

According to GLOBOCAN 2020, female breast cancer (BC) is the most common type of cancer. In 2020, female BC accounted for nearly 700,000 cancer deaths and 2.3 million new cases worldwide (1). Breast cancer is highly heterogeneous, and its treatment classically depends on its clinical status (early stage, locally advanced or metastatic), histological characteristics and biomarker profile (2). Histologically, breast cancers can be classified as sarcomas or carcinomas depending on whether they originate from connective tissue or epithelial cells. In turn, carcinomas are classified as carcinomas *in situ* if they have not invaded other tissues, or as invasive carcinomas if they have invaded adjacent tissues or other organs of the body. Both *in situ* and invasive carcinomas are found in the lobules and ducts (lobular carcinoma *in situ*, LCIS; ductal carcinoma *in situ*, DCIS; invasive lobular carcinoma, ILC; and invasive ductal carcinoma, IDC) (2). The presence or absence of estrogen receptor (ER), progesterone receptor (PR), and human epidermal growth factor receptor 2 (HER2) molecularly determines breast cancer subtypes (3). Estrogen receptor positive (ER+) tumors require estrogen to subsist and grow. PR expression, in turn, is estrogen dependent. Therefore, ER+ and/or PR+ tumors are amenable to endocrine therapies targeting estrogen biosynthesis or estrogen receptors (4, 5). Likewise, HER2 amplification or overexpression determines the use of HER2-targeted therapy (6). Breast cancer can be classified into luminal A, luminal B, HER2-enriched and triple-negative breast cancer (TNBC), also known as basal-like. The luminal A subtype is characterized as ER+ and/or PR+ but HER2−, the luminal B subtype is ER+ and/or PR+/HER2+, the HER2-enriched subtype is characterized by overexpression of HER2 and ER−/PR−, and TNBC is negative for ER, PR and HER2 expression (2, 3, 7, 8). In addition, six TNBC subtypes have been identified: basal-like 1 (BL1), basal-like 2 (BL2), mesenchymal (M), mesenchymal stem-like (MSL), immunomodulatory (IM), and luminal androgen receptor (LAR) (9). In principle, the stratification of patients allows the establishment of treatments adapted to each subtype of breast cancer (2, 10) (**Figure 1**).

Despite efforts, 700,000 women died from breast cancer in 2020 (1). Apparently, prognostic markers currently used in the clinic, such as ER and HER2 status, histologic subtype, size, lymph node status and Nottingham grade, as well as current predictive tests such as germline *BRCA* status, tumor *PIK3CA* mutation status and programmed death-ligand 1 (PDL-1) status, are not sufficient to determine the most appropriate treatment (11). Relapses are the main obstacle faced by clinicians and are mainly due to the development of resistance to the drugs administered. However, there are still insufficient data to determine whether resistance develops after exposure to the drug or whether resistant cells are present from the onset of the disease, preceding antineoplastic treatments (12). This has prompted the search for biomarkers related to different characteristics of breast cancers, such as genetic alterations, epigenetic reprogramming, tumor-promoting inflammation and immune evasion, dysregulation of cell metabolism, or changes in the microbiota, among others (13, 14). Over the past 50 years, efforts have been made to identify genetic and post-transcriptional alterations, changes in protein expression, and more recently, metabolic changes and even immunological or microbial changes. These changes can be detected at the single molecule or pathway level and can serve as markers for diagnosis and/or discovery of personalized therapies. The detection of these modifications is made possible by technologies known as “omics”, such as genomics, transcriptomics, proteomics or metabolomics, or more specific ones such as epigenomics, glycomics or lipidomics, among others. The identification of new biomarkers might allow more precise, and personalized therapeutic decisions in both primary and recurrent cancers (**Figure 1**).

Genomics collectively characterizes and quantifies all the genes of an organism. Genomic analysis includes techniques such as microarrays, gene expression profiling, serial analysis of gene expression (SAGE), comparative genomic hybridization (CGH), array-based CGH (array-CGH), whole genome amplification (WGA), and high-throughput sequencing or next-generation sequencing (NGS). Different genomic studies have focused on the search for markers with predictive or prognostic value, for patient stratification and for determining the appropriate therapy (15, 16). Transcriptomics studies the set of all RNA transcripts of an organism. The most commonly used techniques to study the transcriptome are microarrays and RNA sequencing (RNA-seq) using high-throughput sequencing or NGS. Transcriptomic analysis allows the measurement of differential gene expression, often presented as hierarchical clustering (17, 18). Proteomics detects and quantifies the presence of proteins produced or modified by an organism. Proteomic analysis is performed by using separation techniques (gas chromatography, liquid chromatography, ultra-high performance liquid chromatography, capillary electrophoresis) combined with mass spectrometry (MS, resulting in GC-MS, LC-MS, UPLC-MS, CE-MS, respectively), nuclear magnetic resonance spectroscopy (NMR), reverse phase protein arrays (RPPA), and sequential windowed acquisition of all theoretical fragment ion mass spectra (SWATH-MS) (19). Glycomics provides insight into the biological significance of N-glycosylation of plasma proteins in cancer (20). Metabolomics approaches based on NMR, LC-MS, GC-MS and desorption electrospray ionization mass spectrometry imaging (DESI-MSI) enable the linking of genotype and phenotype thanks to the knowledge generated by dynamic metabolism (21, 22). Epigenomics (study of gene modifications through the aggregation of chemical compounds, with no changes in the DNA sequence), lipidomics (study of lipids in the biological system), and microbiomics (study of microorganisms present in the human body) are branches that use some of the mentioned above.

Due to the complexity that governs carcinogenesis and tumor progression, recent years have seen efforts to integrate data from different omics into a computational approach that allows for more complex reconstruction of biochemical connections (23). This review aims to summarize the existing knowledge on the advances made thanks to omics studies in human breast cancer. In particular, we focus on 1) the discovery of markers that can be used for diagnosis, 2) molecular and/or signaling pathway alterations in onset, progression, and metastasis, and 3) resistance to therapies and attempts to establish personalized treatments (**Figure 1**).

## 2 Implication of omics in the identification of cancer-specific and prognostic biomarkers

A cancer biomarker, found in tissues or body fluids, is used to detect the presence of cancer (differences between tumor and healthy samples or between tumor subtypes) and provides information on prognosis/prediction, cancer progression, and cancer medicine/treatment guidance, among others (24). In this review, we provide an overview of cancer-specific molecules and/or pathways that have been identified as biomarkers in breast cancer using different omics technologies.

### 2.1 Diagnostic biomarkers

One of the first uses of omics has been to identify biomarkers that differentiate tumor tissue from healthy tissue, as well as to identify biomarkers specific to different subtypes of breast cancer. In addition, the last decade has seen efforts to develop minimally invasive techniques to predict breast cancer subtypes, such as identifying biomarkers in human plasma, saliva, and urine.

The presence, copy number alterations, mutations, or amplifications of various genes are markers of breast cancer in tissue. **Table 1** summarizes the most commonly mutated genes (27, 44). Copy number alterations have been observed in *PIK3CA*, *ERBB2*, *TP53*, *MAP2K4*, *MLL3*, *CDKN2A*, *PTEN*, and *RB1* (44). Approximately 35-40% of primary breast cancers harbor mutations in *TP53* and *PIK3CA*, as well as amplification of *ERBB2*, *FGFR1*, and *CCND* (~15%), and mutations in *MUC16*, *AHNAK2*, *SYNE1*, *KMT2C*, *AKT1*, and *GATA3* genes (10%) (25, 45). In 2012, the Cancer Genome Atlas Network (CGAN) identified novel significantly mutated genes (SMGs), including *TBX3*, *RUNX1*, *CBFB*, *AFF2*, *PIK3R1*, *PTPN22*, *PTPRD*, *NF1*, *SF3B1*, and *CCND3* (26).

**Table 1:** Diagnostic molecular biomarkers of breast cancer detected by different omics.

Omic	Molecular Biomarkers	Sample	REF
**Genomic**	*PIK3CA*, *TP53*, *GATA3*, *PTEN*, *AKT1*, *CDH1*, *ARID1B*, *CASP8*, *BRCA1/2*, *RB1*, *MLL3*, *MAP3K1*, *MAP3K13*, *NCOR1*, *SMARCD1*, *CDKN1B*, *TBX3*, *RUNX1*, *CBFB*, *AFF2*, *PIK3R1*, *PTPN22*, *PTPRD*, *NF1*, *SF3B1*, *CCND1*, *CCND3, MAP2K4*, *CDKN2A, BARD1, CHECK2, CTLA4, CYP19A1, FGFR1/2, H19, LSP1, MUC16*, *AHNAK2*, *SYNE1, CDKN1B, RUNX1*, *CBFB*, *AFF2*, *PTPN22*, *PTPRD*, *CCND3, MRE11A, RAD51C, STK11, TERT, TOX3, XRCC2, XRCC3, ERBB2, ARID5B, CTCF, HDAC9, KDM5B, NCOR2, SETD1A, SXL2, ARID1A, CTNND1, NUP107, CHD8, FANC1, CHD9, KEAP1, PCDH18, LAMA2, HDAC9, ARFGEF1, MILT4, FOXO3, GPS2.*	Tissue	(20, 21, 25, 26, 27)
**Glycomic**	H6N5F1L3, H6N5L2E1, H7N6F1L1E3, H6N5F1S3, Vimentin, keratin 7, enolase 2, pyruvate kinase isozyme M 2 (PKM2), protein disulfide isomerase (PDI) A6, TP and voltage-dependent anion selective channel protein in breast cancer	Tissue	(28, 29, 30, 31)
**Metabolomic**	N1-Ac-SPD, N8-Ac-SPD	Saliva	(32)
**Metabolomic**	Acetone, 3-hexanone, 4-heptanone, 2-methyl-5-(methylthio)-furan and acetate	Urine tissue	(33)
**Metabolomic Proteomic**	taurocholate, taurochenodeoxycholate, glycocholate, allantoin, taurodesoxycholate, glycodesoxycholate, ursodeoxycholate, mannose and fructose, tyramine O-sulfate, N-formylphenylalanine, dopamine 4-sulfate, glycerol, glycerol 3-phosphate, GOT1, LDHB, GSS, GPX3	Plasma	(34)
**Metabolomic**	Lactate, lysine, alanine, pyruvate and glucose	Serum	(35)
**Lipidomic**	S1P, ceramides, sphingomyelin phosphodiesterase, palmitate-containing phosphatidylcholines	Tissue	(36, 37, 38)
**Lipidomic**	Lysophosphatidylcholine, lysoPC a C16:0, PC ae C42:5 and PC aa C34:2	Plasma	(39)
**Microbiomic**	*Thermus scotoductus*, *E. coli*, *Bacillus cereus*, *Shewanella*, *Corynbacterium, Bacillus, Staphylococcus, Enterobacteriaceae, Comamondaceae, Bacteroidetes*, *Mycobacterium fortuitum, Mycobacterium phlei, Fusobacterium*, *Atopobium*, *Gluconacetobacter*, *Hydrogenophaga* and *Lactobacillus*	Tissue	(40, 41, 42, 43)

A proteomic analysis has identified canonical up- and downregulated pathways in breast cancer. Pathways upregulated in breast cancer include glycolysis; metabolic pathways such as pyruvate fatty acid, arginine and proline, and valine, leucine and isoleucine catabolism; protein ubiquitination; RhoA, PI3K/AKT, ILK, 14-3-3-mediated, RAN, aryl hydrocarbon receptor, integrin, clathrin-mediated endocytosis, IGF-1, VEGF, EIF2, actin cytoskeleton, ERK5, GABA receptor, and HER-2 signaling pathways; NRF2-mediated response to oxidative stress; Rho-mediated regulation of actin-based motility; and LPS/IL-1-mediated inhibition of RXR function. Downregulated pathways include the citrate cycle, acute phase response signaling, P53 signaling, primary immunodeficiency signaling, urea cycle and amino group metabolism, Cdc42 signaling, glyoxylate and dicarboxylate metabolism, and autoimmune thyroid disease signaling (46).

A fucosylated triantennary glycan containing three α2-3 sialic acids (also called H6N5F1L3) and a non-fucosylated triantennary glycan containing a combination of α2-3 and α2-6 sialic acids (H6N5L2E1) are found at lower levels in breast cancer patient samples (28), although other studies have reported conflicting data for H6N5F1L3 (47). In addition, a fucosylated tetraantennary glycan containing a combination of α2-3 and α2-6 sialic acids (H7N6F1L1E3) is significantly elevated in breast cancer patients (28). Similarly, elevated levels of other trisialylated triantennary fucosylated glycans (termed H6N5F1S3, consisting of H6N5F1E3, H6N5F1L3, H6N5F1L2E1, H6N5F1L2E1, H6N5F1L1E2, and H6N5F1E3) have been observed (28, 30). Glycomic analysis have identified seven glycosylated proteins with *O*-linked β-D-N-acetylglucosamine (*O*-GlcNAc), an important post-translational modification involving reversible and highly dynamic covalent binding of β-N-GlcNAc to Ser/Thr residues in proteins (48), such as vimentin, keratin 7, enolase 2, pyruvate kinase isozyme M 2 (PKM2), protein disulfide isomerase (PDI) A6, TP, and voltage-dependent anion selective channel protein in breast cancer (31) (**Table 1**).

Eight pathways have been implicated in breast cancer: protein digestion and absorption, central carbon metabolism in cancer, neuroactive ligand receptor interaction, ABC transporters, mineral uptake, inositol phosphate metabolism, glutathione metabolism, and cysteine and methionine metabolism (49). A combinatorial study of metabolomic and proteomic profiles in human plasma samples has identified metabolic signatures for breast cancer diagnosis. The most abundant metabolites in breast cancer patients include mainly primary and secondary compounds of bile acid metabolism, compounds of fructose, mannose, galactose, tyrosine and glycerolipid metabolism. Critical metabolic pathways in breast cancer include alanine, aspartate and glutamate pathways, glutamine and glutamate metabolic pathways, and arginine biosynthesis pathways. The specific metabolites are listed in **Table 1**. In addition, thanks to the combined study with the proteome, aspartate aminotransferase (GOT1), L-lactate dehydrogenase B-chain (LDHB), glutathione synthetase (GSS) and glutathione peroxidase 3 (GPX3) have been found to be closely involved in these metabolic pathways (34).

On the other hand, metabolomic analysis of polyamines in saliva before and after surgical treatment has shown a decrease in N1-Ac-SPD and an increase in N8-Ac-SPD in patients after surgical treatment. Thus, the ratio (N8-Ac-SPD)/(N1-Ac-SPD+N8-Ac-SPD) could be an index of health status after surgical treatment (32) (**Table 1**). A metabolomic study of urine and breast tissue samples has identified dysregulation of lactate, valine, aspartate and glutamine pathways in breast cancer. In addition, five metabolites (acetone, 3-hexanone, 4-heptanone, 2-methyl-5-(methylthio)-furan, and acetate) allow correlation between urine and tissue samples (33). Inositol triphosphate receptor (IP3R) type 2 and 3 expression is increased in breast tumor tissue compared to adjacent healthy tissue. Increased lipoproteins, increased levels of metabolites such as lactate, lysine and alanine, and decreased levels of pyruvate and glucose in the serum of patients with high IP3R expression compared to healthy individuals (35).

The combination of metabolic profiling with tissue protein expression increases the accuracy in characterizing breast cancer patients (35). Lipidomic analyses have shown that the levels of sphingosine-1-phosphate (S1P), ceramides, and other sphingolipids are significantly higher in breast tumors than in normal breast tissue (36). In addition, sphingomyelin phosphodiesterase (SMase), which converts sphingomyelin to ceramide phosphate, is downregulated in 60% of breast tumors (37). An increase in fatty acids such as palmitate-containing phosphatidylcholines (PC) has also been found, especially in ER− and grade 3 tumors compared to healthy breast tissue. Phospholipids may have diagnostic potential as they have been associated with cancer progression and patient survival (38). When plasma samples from breast cancer patients and healthy controls were compared, significantly lower levels of lysophosphatidylcholines (LysoPC) and higher levels of sphingomyelins have been observed in plasma samples from cancer patients. In addition, three metabolites (LysoPC a C16:0, PC ae C42:5 and PC aa C34:2) differentiate breast cancer patients from healthy controls (39) (**Table 1**).

Lastly, the bacteria found on the skin have direct access to the breast ducts through the nipple, so the breast tissue contains a wide variety of bacteria, such as *Staphylococcus epidermidis* and *Micrococcus luteus* (50, 51). Different species of bacteria perform different functions. For example, *Lactobacillus* triggers protective mechanisms that include immune activation, competitive inhibition of pathogenic strains, and synthesis of signaling intermediates. In contrast, *E. coli* and Staphylococcus induce DNA damage (40). Changes in the microbiota of breast and intestinal tissues have been associated with the development of breast cancer (50). Several studies even describe correlations between dysbiosis of the tissue microbiome and the development of breast cancer. When characterizing the microbiome of tumor tissue and adjacent non-tumor tissue from different patients, a higher abundance of taxa belonging to the phylum Actinobacteria was observed in the non-tumor samples. In contrast, Firmicutes and Alpha-Proteobacteria were significantly overrepresented in tumor tissue (52). Healthy individuals show a significantly higher abundance of *Lactobacillus, Thermoanaerobacterium thermosaccharolyticum*, *Candidatus Aquiluna* sp.*, IMCC13023*, *Anoxybacillus, Leuconostoc*, *Lactococcus, Geobacillus, Methylobacterium, Turicella otitidis* (40), *Prevotella*, *Lactococcus*, *Streptococcus*, *Corynebacterium* and *Micrococcus* (41). In cancer patients, there is an abundance of *Thermus scotoductus*, *E. coli*, *Bacillus cereus*, *Shewanella*, *Corynbacterium* (40), *Bacillus*, *Staphylococcus*, Enterobacteriaceae, Comamondaceae, Bacteroidetes (41), *Mycobacterium fortuitum*, and *Mycobacterium phlei* (42). The breast tissue microbiome of women with enrichment in lower abundance taxa, including the genera *Fusobacterium*, *Atopobium*, *Gluconacetobacter*, *Hydrogenophaga* and *Lactobacillus*, compared to that of women with benign breast disease (43) (**Table 1**).

### 2.2 Stratification biomarkers

Genomic alterations have been found in the different molecular subtypes of primary breast cancer. In general, approximately 5-10% of breast cancers are hereditary, mostly due to pathogenic variants or mutations in the *BRCA1* and *BRCA2* genes (53). Mutations in *BRCA1* are associated with ER− and PR− tumors (54, 55), while mutations in *BRCA2* are associated with ER+ and PR+ tumors (56). SMGs are more diverse and recurrent in both luminal subtypes, particularly in luminal A (26), and the heat shock protein (HSP) family has been specifically associated with different cancer types (18). In the luminal subtype A, the most common SMG is *PIK3CA* (45%), followed by *MAP3K1*, *GATA3*, *TP53*, *CDH1*, and *MAP2K4* (26). Copy number gains of *CCND1*, *FGF3*, and *FGFR1* have also been identified (25). The heat shock protein (HSP) genes *DNAJB4*, *DNAJC18*, *HSPA12A*, *HSPA12B*, *HSPB2*, *HSPB6*, *HSPB7*, *CRYAB*, and *SACS* are downregulated, whereas *DNAJC5B*, *DNAJB13*, *DNAJC1*, *DNAJC22*, *HSPB1*, *HSPA6*, and *DNAJC12* are upregulated (18). The luminal subtype B is characterized by *TP53* and *PIK3CA* SMGs (29% each) (26), as well as increased copy number of *CCND1*, *FGF3* and *FGFR1* (25); downregulation of HSP genes such as *DNAJB4*, *DNAJC18*, *HSPA12A*, *HSPA12B*, *HSPB2*, *HSPB6*, *HSPB7*, *CRYAB* and *SACS* and upregulation of *DNAJC5B*, *DNAJB13*, *DNAJC1*, *DNAJC22*, *HSPB1*, *HSPA6*, *CCT5*, *CCT3*, *HSPE1*, *DNAJC9*, *HSPD1*, *DNAJC12*, *DNAJA4*, *HSPH1*, *CCT2*, and *DNAJA3* (18). The HER2-enriched subtype presents with HER2/*ERBB2* amplification (80%), high frequency of mutations in *TP53* (72%) and *PIK3CA* (39%) (26). *PTEN* and *INPP4B* have also been identified as genes of interest. Deletions in *PPP2R2A*, *MTAP*, and *MAP2K4* genes have been reported (45). Among the HSP genes, *CRYAB*, *SACS*, *DNAJB4*, *DNAJC18*, *HSPA12A*, *HSPA12B*, *HSPB2*, *HSPB6*, *HSPB7*, *HSPB8*, *DNAJC5G* and *BBS12* are downregulated, while the upregulated genes are *DNAJC5B*, *DNAJB13*, *DNAJC1*, *DNAJC22*, *HSPB1*, *CCT5*, *CCT3*, *HSPE1*, *DNAJC9*, *HSPD1*, *DNAJA4*, *HSPH1*, *HYOU1*, *DNAJB11*, *CCT6A* and *DNAJB3* (18). Finally, mutations in *TP53* are observed in 80% of TNBC cases, followed by alterations in *PIK3R1* and *NF1* (26). *INPP4B* is another gene of interest in TNBC, and deletions in *PPP2R2A*, *MTAP* and *MAP2K4* genes have also been reported (45). Among the downregulated HSP genes in TNBC, we found *DNAJB4*, *DNAJC18*, *HSPA12A*, *HSPA12B*, *HSPB2*, *HSPB6*, *HSPB7*, *HSPB8*, *DNAJC12* and *DNAJC27*. As for the upregulated genes, *DNAJC5B*, *HSPA6*, *CCT5*, *CCT3*, *HSPE1*, *DNAJC9*, *HSPD1*, *HYOU1*, *DNAJB11*, *CCT6A*, *HSPA5*, *HSPA14*, *CRYAA*, *DNAJC2* and *DNAJC6* were found (18) (**Table 2**).

**Table 2:** Stratification biomarkers of breast cancer detected by different omics.

Omic	Biomarkers	Sample	Subtype of BC	REF
**Genomic**	*BRCA1*, miR-206	Tissue	ER−	(54, 57)
**Genomic**	*BRCA1*	Tissue	PR-	(47)
**Genomic**	*BRCA2*	Tissue	ER+, PR+	(56)
**Genomic**	*BRCA1, INPP4B*, *PPP2R2A*, *MTAP, MAP2K4 TP53, PIK3R1*, *NF1, DNAJC12, DNAJC27, DNAJC2*, *DNAJC6, DNAJC9, DNAJB11, CCT5*, *CCT3*, *CCT6A, HSPE1*, *HSPD1, HYOU1*, *HSPB8, HSPA6, HSPA5*, *HSPA14*, *CRYAA*		TNBC	(18, 25, 26, 45, 54, 55)
**Genomic**	*PTEN, INPP4B*, *PPP2R2A*, *MTAP, MAP2K4*, HER2, *ERBB2*, *TP53, PIK3CA, CRYAB*, *SACS, HSPB1, HSPB8, DNAJC5G, DNAJB13*, *DNAJC1*, *DNAJC22, DNAJA4, DNAJC9, DNAJB11, DNAJB3, BBS12, CCT5*, *CCT3*, *HSPE1*, *HSPD1, HSPH1, HYOU1*, *CCT6A*	Tissue	HER2-enriched	(13, 21, 23)
**Genomic**	*PIK3CA*, *MAP3K1*, *GATA3*, *TP53*, *CDH1*, *MAP2K4, CCND1*, *FGF3*, *FGFR1, CRYAB*, *SACS, DNAJC12, DNAJB13*, *DNAJC1*, *DNAJC22, HSPB1, HSPA6*	Tissue	Luminal A	(18, 25, 26)
**Genomic**	*TP53*, *PIK3CA, CCND1*, *FGF3*, *FGFR1, CRYAB*, *SACS, HSPB1, HSPA6, CCT5*, *CCT3*, *HSPE1*, *DNAJC9*, *HSPD1, DNAJC12, DNAJA4, DNAJB13*, DNAJC1, *DNAJA3, DNAJC22, HSPH1, CCT2*	Tissue	Luminal B	(18, 25, 26)
**Transcriptomic**	miR-21, miR-200c, miR-361-5p, miR-374a, miR-93, miR-182, miR-183, miR-210, miR-221, miR-7b, miR-125b, miR-127-3p, and miR-320	Tissue	DCIS	(57)
**Transcriptomic**	miR-375, miR-182, miR-183, miR-96, miR-203, miR-425-5p, miR-565	Tissue	LCIS	(57)
**Proteomic**	PPIaseB, Rho-GDI α, TPM4, Thymosin α1, PGRMC1, Liprin-α1, β-arrestin-1, fascin, DAP5, superoxide dismutase, Ral A binding protein, Galectin-1, uridine phosphorylase 2, cellular retinoic acid-binding protein 1, protein S100-A11, nucleoside diphosphate kinase A, α1-antitrypsin	Tissue	HR+	(58)
**Proteomic**	HSP90α, laminin, GSTP1, FASN, HSP27, PGK1, GLO, CK19, HNRNPH1, BiP, RKIP CK7, GAPDH, PGK1, FUT8, HEXA, HEXB, MAN2B2, MAN1B1, MAN2A1, GALNT 2,3,6, ADH, ALDH, ACAD, PYCR1,2, PYCRL, PRODH, HNMT, KMO	Tissue	HER2-enriched	(58, 59)
**Proteomic**	STAT1, PTEN, pMAPK, P38, P27, P21, MASPIN, CD10, FAK, EGFR, Caveolin, CD74, CK14, RCL1, MCM complex proteins, DNA polymerases, DNA damage response proteins, CDK1, CDK2, CDK6, PCNA, PTEN	Tissue	TNBC	(58)
**Proteomic**	FBP2, FBP1, NDUF, UQCR, SDH, COX subunits, ATP5, ATP6 subunits, CA1, CA2	Tissue	Luminal	(59)
**Proteomic**	EZH2	Tissue	DCIS	(15)
**Genomic Transcriptomic Proteomic**	CDH1, TGFBR2, IL11RA, TNFRSF17, CCL15, CCL14, CCR2, CD27, XCL2, IFNAR2 CD40LG, PDCD1 (PD-1), CD274 (PD-L1), CTLA4	Tissue	ILC	(60)
**Metabolomic**	β-alanine, xanthine, isoleucine, glutamate, taurine	Tissue	ER+	(61)
**Metabolomic**	Glycochenodeoxycholic acid, alanine, LysoPC (16:1), valine, 2-octenedioic acid	Plasma	ER+	(60)
**Metabolomic**	Carnitine, LysoPC (20:4), proline, valine, 2-octenedioic acid	Plasma	HER2+	(60)
**Metabolomic**	L-Tryptophan, LysoPC(14:0), Glycoursodeoxycholic acid, Lysophosphoethanolamines (LysoPE)(18:2)	Plasma	Luminal A	(62)
**Metabolomic**	LysoPE(18:2), LysoPE(18:1(11Z/9Z)), LysoPC(20: 3), Biliverdin, LysoPE(16:0)	Plasma	Luminal B	(62)
**Metabolomic**	LysoPE(18:1(11Z)/9Z), LysoPC(0:0/16:0), Biliverdin, L-Tryptophan, LysoPE(18:2)	Plasma	HER2-enriched	(62)
**Metabolomic**	LysoPE(18:1(11Z)/9Z), LysoPC(0:0/16:0), Biliverdin, L-Tryptophan, LysoPE(18:2) in HER2+; and L-Tryptophan, LysoPC(16:0/0:0), LysoPE(18:1(11Z)/9Z)	Plasma	TNBC	(62)
**Lipidomic**	taurine (m/z 124.0068), uric acid (m/z 167.0210), ascorbic acid (m/z 175.0241) and glutathione (m/z 306.0765)	Tissue	IBC	(61)
**Lipidomic**	fatty acids (341.2100 and 382.3736 m/z) and glycerophospholipids (PE (P-16:0/22:6, m/z 746.5099, and PS (38:3), m/z 812.5440)	Tissue	DCIS	(61)
**Lipidomic**	glycerol-3-phosphate acyltransferase	Tissue	HR-associated	(63)
**Lipidomic**	GM2	Plasma	ER-	(64)
**Microbiomic**	*Bordetella*, *Campylobacter*, *Chlamydia*, *Chlamydophila*, *Legionella*, and *Pasteurella*	Tissue	Luminal B	(65)
**Microbiomic**	*Arcanobacterium, Bifidobacterium, Cardiobacterium, Citrobacter, and Escherichia*	Tissue	Luminal A	(65)
**Microbiomic**	*Streptococcus*	Tissue	HER2-enriched	(65)
**Microbiomic**	*Aerococcus*, *Arcobacter*, *Geobacillus*, *Orientia*, and *Rothia*	Tissue	TNBC	(65)

DCIS, ductal carcinoma *in situ*; ER, estrogen receptor; HR, hormone receptor; IBC, invasive breast cancer; ILC, invasive lobular cancer; LCIS, lobular carcinoma *in situ*; PR, progesterone receptor; TNBC, triple negative breast cancer.

MicroRNAs (miRNAs) are small non-coding RNAs that modulate gene expression to regulate various cellular processes, including those involved in breast cancer (66, 67). miR-206 is highly expressed in ER− tumors and also targets the ERα receptor, as do miR-221 and miR-222 (57). In addition, DCIS and LCIS are characterized by the upregulated expression of several miRNAs, which are listed in **Table 2** (57).

Through an integrated study combining genomics and epigenomics, pathways unique to TNBC and non-TNBC were identified. The most significant pathways for TNBC are retinal biosynthesis, BAG2, LXR/RXR, EIF2, and P2Y purinergic receptor signaling pathways, whereas in non-TNBC they are UVB-induced MAPK, PCP, endothelial apelin, endoplasmic reticulum stress, and host viral egress mechanisms (68). Based on the genomic and transcriptomic landscape, Xie et al. have established a new classification of immune subtypes of ER+/PR−/HER2− breast cancer, termed clusters 1 to 5. Cluster 1 is characterized by an activated but suppressive immune microenvironment, immune infiltration, increased stromal content, and an elevated TGF-β response signature. Cluster 2 has an inactivated immune phenotype. Cluster 3 has an activated immune phenotype enriched in innate, adaptive, and immunosuppressive cells, as well as interferon (IFN)-γ response, inflammation, macrophage upregulation, and cytolytic signatures. Cluster 4 is characterized by an immunologically inactive phenotype and low infiltration of the microenvironment. Finally, cluster 5 lacks immunologic properties but presents a phenotype associated with hormonal responses (69) (**Table 2**).

Several studies have investigated the classification of breast cancers using proteomic technologies. By analyzing differential protein expression in tissues, they identified the expression of proteins that characterize luminal subtypes, HER2-enriched breast cancers, and TNBC. In addition, proteomic profiling of these three breast cancer types revealed functional differences. Cytoskeletal remodeling as well as alterations in the cell adhesion process are found in all BC types. Luminal tumors are characterized by increased “energy metabolism” as indicated by an elevated expression of key proteins in gluconeogenesis, electron transport chain and ATP synthase complex. “Immune response” is altered in both luminal A and luminal B subtypes, while “cell cycle regulation” is important in luminal B tumors. On the other hand, luminal tumors show decreased expression of proteins related to metabolic pathways, including glycolysis, serine synthesis, and glutamine consumption. The HER2-enriched subtype is characterized by decreased “amino acid and energy metabolism”, reduced “cellular community” and increased “glycan biosynthesis and metabolism”. Finally, the TNBC subtype is characterized by increased “replication and repair”, “cell growth and death” and “translation” pathways. Other relevant processes are “immune response” and “blood coagulation”. The most relevant proteins involved in these pathways are listed in **Table 2** (58, 59, 70). Besides that, the expression of the protein enhancer of zeste homolog 2 (EZH2) is elevated in premalignant atypical ductal hyperplasia (ADH) and even higher in DCIS compared to normal epithelium (15).

Luminal tumors have been classified into 3 proteomic clusters. Luminal cluster-1 is enriched for RNA processing and splicing processes but depleted for immune-related proteins including the ones involved in antigen processing and presentation, and type I and type II IFN signaling. Luminal cluster-2 is enriched for stromal proteins and extracellular matrix (ECM) components. Luminal cluster-3 has high expression of proteins for DNA replication, cell cycle, response to DNA damage, and immune response, while depleted for ECM components, blood coagulation, epithelial cell differentiation, and response to estrogen and steroid hormones compared to luminal clusters-1 and -2. In addition, significantly higher expression of Ki67 is found in luminal cluster-3 compared to luminal cluster-1 and -2 (71). On the other hand, 4 TNBC subgroups have been identified according to 4 proteomic clusters. TNBC cluster-1 has the most favorable survival and is characterized by immune response, antigen processing and presentation, and IFN type I and II signaling processes. TNBC cluster-2 has intermediate survival and is enriched for ECM components, coagulation, and humoral immune response processes. TNBC cluster-3 has intermediate survival and is enriched for lipid metabolism, catabolism, and oxidation-reduction processes. TNBC cluster-4 exhibits the poorest survival and is enriched for DNA replication and cell cycle proteins (71).

A comprehensive genomic, transcriptomic and proteomic analysis of patients with ILC has identified mutations in cadherin-1 (CDH1) and the phosphatidylinositol 3- kinase (PI3K) pathway as the most common molecular alterations in ILC. In addition, two major subtypes of ILC have been identified: an immune-related (IR) subtype and a hormone-related (HR) subtype: The IR subtype is characterized by upregulation of PD-L1 mRNA, PD-1 and CTLA-4, increased sensitivity to DNA damaging agents, and upregulation of lymphoid signaling molecules at the mRNA level (TGFBR2, IL11RA, TNFRSF17, CCL15, CCL14, CCR2, CD27, XCL2, IFNAR2, and CD40LG). In addition, the IR subtype shows upregulated genes in the cytokine-cytokine receptor interaction pathway, suggesting alterations in the composition or functional activity of immune cells within these tumors. Interestingly, the negative regulators of the immune response PDCD1 (PD-1), CD274 (PD-L1) and CTLA4 are expressed at higher mRNA levels in the IR subtype. The related HR subtype is associated with epithelial-to-mesenchymal transition (EMT). Moreover, the HR subtype shows higher levels of estrogen receptors (ESR1) and progesterone receptors (PGR) and upregulation of cell cycle genes and estrogen receptor (ER) target genes (60) (**Table 2**).

Differential metabolites have been identified when comparing HER2+ with HER2− patients, as well as when comparing ER+ with ER− patients. Plasma samples from HER2+ patients are characterized by increased aerobic glycolysis, gluconeogenesis and fatty acid biosynthesis and decreased Krebs cycle. Specifically, HER2+ is characterized by overexpression of carnitine, LysoPC (20:4), proline, valine, and 2-octenedioic acid. Strong metabolic differences correlate with hormone receptor status. Plasma samples from ER+ patients reflect increased alanine, aspartate, and glutamate metabolism, decreased glycerolipid catabolism and increased purine metabolism. ER+ is characterized by overexpression of glycochenodeoxycholic acid and decreased expression of alanine, LysoPC (16:1), valine, and 2-octenedioic acid. Many glycolytic and glycogenolytic intermediates, components of the glutathione (GSH) pathway, the oncometabolite 2-hydroxyglutarate (2-HG), and the immunomodulatory tryptophan metabolite kynurenine are elevated in ER− compared to ER+ cancers (72, 73). Using deep learning techniques, metabolites, and pathways have been identified that can discriminate between ER+ and ER− patient samples. Among the identified metabolites, five have been proposed as breast cancer biomarkers: β-alanine, xanthine, isoleucine, glutamate, and taurine (49). Studies performed on plasma samples from breast cancer patients have allowed the identification of specific metabolomic profiles for each cellular subtype: L-tryptophan, LysoPC(14:0), glycoursodeoxycholic acid, lysophosphoethanolamine (LysoPE)(18:2) in luminal A; LysoPE(18:2), LysoPE(18:1(11Z/9Z)), LysoPC(20: 3), biliverdin, LysoPE(16:0) in luminal B; LysoPE(18:1(11Z)/9Z), LysoPC(0:0/16:0), biliverdin, L-tryptophan, LysoPE(18:2) in HER2+; and L-tryptophan, LysoPC(16:0/0:0), LysoPE(18:1(11Z)/9Z) in TNBC (62) **Table 2**. When plasma samples from patients with mutated *BRCA1* and non-mutated *BRCA1* were compared, the levels of adenine, N6-methyladenosine, and 1-methylguanine were found to be significantly lower in patients with *BRCA1* mutations (74).

The lipidomic profiles of invasive breast cancer (IBC), DCIS and benign surrounding tissue (BAT) have been investigated. IBC is characterized by the presence of polyunsaturated fatty acids, deprotonated glycerophospholipids and sphingolipids. IBC can be distinguished from BAT by the presence of highly saturated lipids and antioxidant molecules. DCIS differs from IBC by lipids associated with cell signaling and apoptosis (61). Lipidomics have allowed the identification of glycerol-3-phosphate acyltransferase (GPAM), an enzyme involved in triacylglycerol and phospholipid biosynthesis, which shows increased cytoplasmic expression in HR-associated breast cancer and improved OS (63) (**Table 2**).

Finally, Benarjee et al. have identified a local microbial signature associated with each type of breast tumor. *Actinomyces*, *Bartonella*, *Brevundimonas*, *Coxiella*, *Mobiluncus*, *Mycobacterium*, *Rickettsia*, and *Sphingomonas* are common in all types of breast cancer. In the luminal A subtype, *Arcanobacterium*, *Bifidobacterium*, *Cardiobacterium*, *Citrobacter*, and *Escherichia* are detected. *Bordetella*, *Campylobacter*, *Chlamydia*, *Chlamydophila*, *Legionella*, and *Pasteurella* are associated with the luminal B subtype. The HER2-enriched subtype is characterized by the presence of *Streptococcus*, whereas *Aerococcus*, *Arcobacter*, *Geobacillus*, *Orientia*, and *Rothia* are associated to with TNBC (65) (**Table 2**).

### 2.3 Prognostic biomarkers

The various omics technologies have allowed the identification of prognostic biomarkers in both solid and liquid samples. Gene expression analysis have identified genes associated with good and poor prognosis in breast cancer (18). In addition, using data from breast cancer databases (TCGA-BRCA and CMI-MBC), a 45-gene optimal prognostic gene signature has been constructed from genes regulated by tumor-associated macrophages (TAMs). All these genes are listed in **Table 3** (75).

**Table 3:** Prognostic biomarkers of breast cancer detected by different omics.

Omic	Biomarkers	Prognosis	REF
**Genomic**	*HSPA2*, *DNAJB5*, *HSCB*, *HSPA12B*	Good	(18)
**Genomic**	*CCT6A*, *DNAJA2*, *HSPA14*, *CCT7*, *HSPD1*, *CCT2*, *HSPA4*, *DNAJC6*, *CCT5*, *SEC63*, *HSPH1*, *CCT8*, *CCT4*, *HSP90AA1*, *HSPA8*, *DNAJC13*, *HSPA9*, *TCP1*	Poor	(18)
**Genomic**	*CS*, *SMARCE1*, *IGSF9B*, *SYTL4*, *CEMIP*, *EMC2*, *FHL2*, *RAMP3, CISD1, PAICS, TTI2, FIBCD1, ZCCHC9, VAV3, LIMD2, TANK, PAK6, ETFA, PRDM16, ADAM15, NFKBIZ, DDAH1, CC2D1B, SH2B2, ACYP2, ENDOV, KBTBD11, AL162595.1, PCED1B, LYSMD4, TRMT2B, SLC6A9, NOS1AP, LINC01291, PSMB10, RPL12P38, ZNF888, AL391845.1, LINC02585, LINC01431, AC099520.2, CEP95, MIR4713HG, RBM15B, AC061992.2*	-	(75)
**Epigenomic**	Increased levels of DNA methylation, alterations in mRNA expression, hypermethylation of *RASSF1A* and *PITX2*	Poor	(76, 77)
**Proteomic**	BCL2, CDH1, CLDN3, CLDN7, NADP, IDH2 CRABP2, SEC14L2	-	(78, 79)
**Proteomic**	SHMT2, SLC1A5, decorin, endoplasmin, E-cadherin, β-catenin	Poor	(75, 76, 80)
**Proteomic** **Transcriptomic**	FLT1, FADD, ALDOA, CXCL, FGFR1, PLCB3, PPP2R2A, RPA1	Poor	(78)

“-” means without information.

Not only genomic but also epigenomic differences have been found. By applying a powerful integrative network algorithm to paired DNA methylation and RNA-Seq data from ER+ breast cancer and adjacent healthy tissue, it has been shown that increased levels of DNA methylation and alterations in mRNA expression can predict poor prognosis. In particular, epigenetic silencing of WNT signaling antagonists and bone morphogenetic proteins (BMPs) has been observed in both luminal subtypes, but predominantly in luminal B breast cancer (76). Davalos et al. identified hypermethylation of *RASSF1A* and *PITX2* associated with poor prognosis in early stage of breast cancer (77).

Various proteomic analyses have validated proteins related to apoptosis, cell cycle arrest, cell adhesion, cytokeratins, cell metabolism and lipid binding as prognostic biomarkers of breast cancer OS (78, 79). Elevated expression of serine hydroxymethyltransferase 2 (SHMT2) correlates with poor OS and relapse-free survival (RFS), the amino acid transporter ASCT2 (SLC1A5) correlates with poor RFS (80), endoplasmin (HSP90B1) has been associated with distant metastasis and worse OS, and decorin (DCN) has been associated with lymph node metastasis, increased number of positive lymph nodes and worse OS; and (81). Finally, increased levels of E-cadherin and β-catenin correlate with poor survival in invasive breast cancer but not in lobular carcinoma (82) (**Table 3**).

An analysis of breast cancer transcriptomic and proteomic data from the Clinical Proteomic Tumor Analysis Consortium (CPTAC) resource has identified 2 candidates associated with survival: Fms-related receptor tyrosine kinase 1 (FLT1) in TNBC, Fas-associated death domain (FADD) protein with the luminal type, while 8 candidates: Fructose-bisphosphate aldolase A (ALDOA), C-X-C motif chemokine (CXCL)16, fibroblast growth factor receptor 1 (FGFR1), 1-phosphatidylinositol 4,5-bisphosphate phosphodiesterase beta-3 (PLCB3), serine/threonine protein phosphatase 2A 55 kDa regulatory subunit B alpha isoform (PPP2R2A), and replication protein A1 (RPA1) are clearly associated with poor survival in the HER2-enriched type (83). Of note, protein glycosylation correlates with increased tumor burden and poor prognosis in breast cancer (84) (**Table 3**).

## 3 Omics data on the onset of breast cancer

Early-stage breast cancer lesions are so small that there may be insufficient material for analysis and it is difficult to obtain accurate data.

**Figure 2:**
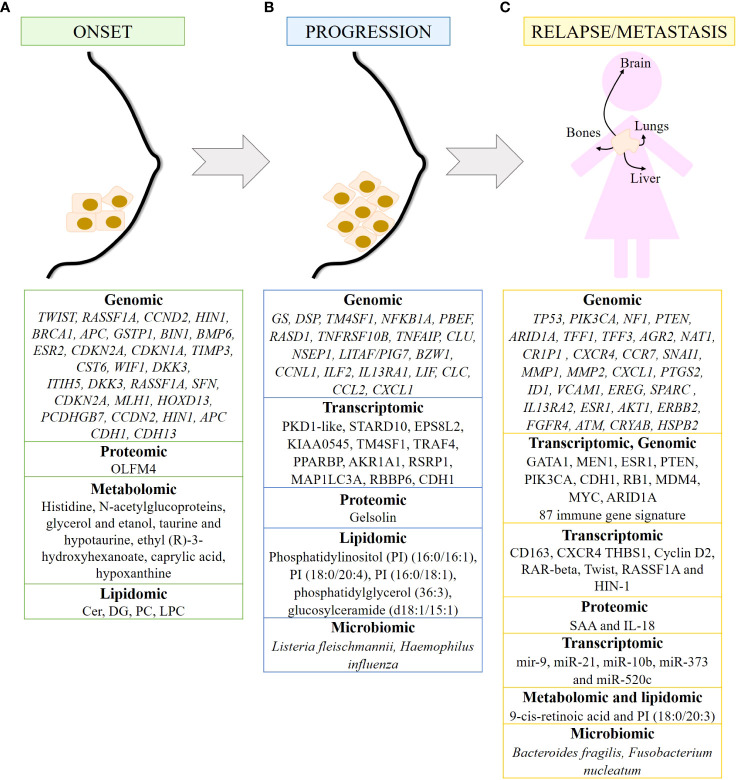
Biomarkers of breast cancer at different time points of tumor development detected by omics. Summary of markers observed in breast cancer onset **(A)**, progression **(B)** and metastasis/relapse **(C)** detected by genomic, transcriptomic, proteomic, metabolomics, lipidomic and/or microbiomic. 87 immune gene signature includes *LAG3, RELB, CCL2, IFNG, MSH6, ZC3HAV1, CD68, ORM1, LYZ, USP14, SLA2, HERC5, LAMP3, NONO, BATF, FCER1G, CCR5, REL, DTX3L, HMGB2, C2, CLEC4E, CLEC4D, CLEC7A, IL12A, CXCL10, CXCL11, RASGRP1, HAVCR2, ICOS, ATRIP, TRIM25, RNF166, CCR8, CSF1, NFAM1, TUBB4B, LYAR, CLEC12A, IL27, PIK3CG, XRCC6, PARP9, DNAJC5, MPEG1, TIFA, TLR1, CD47, EXO1, NCF2, SLAMF7, CTSS, GBP5, GBP4, GBP1, CREG1, RNF19B, RC3H2, RAB14, SYK, ACTR2, KCNAB2, OPTN, DDX58, IL2RA, JAK2, CLEC6A, LYST, CCL25, CCL8, HLADRA, RAB27A, PTK2B, PDCD1LG2, IFI30, TLR6, DSN1, HLA-DOB, CXCR6, TNF, IL10, SERPINA1, GSDMD, TRAF3, IL12RB1, CCL5* and *LIG4*.

The onset of breast cancer is characterized by abnormal paracrine and autocrine signaling, as genes that are highly expressed in healthy breast epithelium are lost in carcinomas, including genes encoding cytokines such as *LIF*, *IL-6*, and *HIN-1*, also known as *SCGB3A1*, and chemokines such as *IL-8*, *GROα*, *GROβ*, and *MIP3α*, also known as *CCL20* (85). In addition, genes silenced by hypermethylation have been identified as responsible for mammary carcinogenesis, including *TWIST, RASSF1A*, *CCND2*, *HIN1*, *BRCA1*, *APC*, *GSTP1*, *BIN1*, *BMP6*, *ESR2*, *CDKN2A*, *CDKN1A*, *TIMP3* and *CST6*, as well as the WNT-negative regulators *WIF1* and *DKK3* (77). In addition, two methylated modifications (H3K9 me2 and me3) of the DNA packaging protein histone H3 decrease during cancer transformation, and the demethylase KDM3A/JMJD1A gradually increases (86). On the other hand, methylated genes such as *ITIH5*, *DKK3*, *RASSF1A*, *SFN*, *CDKN2A*, *MLH1*, *HOXD13* and *PCDHGB7* have been proposed as potential markers for early detection of breast cancer. Hypermethylation of *RASSF1A*, *CCDN2*, *HIN1* and *APC* correlates mainly with HR+ breast cancer, whereas hypermethylation of *CDH1* and *CDH13* is more frequent in TNBC patients (77). Furthermore, the expression of olfactomedin-4 (OLFM4) is higher in non-invasive breast tumors than in invasive breast cancer. Therefore, OLFM4 may also be a biomarker for early breast cancer (87) (**Figure 2A**).

Alterations in circulating metabolites have been identified in premenopausal women that may predict the development of breast cancer. In fact, ten metabolites listed in **Figure 2A** have been associated with breast cancer risk (88). Metabolomic signatures of the taurine and hypotaurine pathways and the alanine, aspartate and glutamate pathways obtained from the plasma of breast cancer patients are critical for early diagnosis (89). In addition, six metabolites and eight metabolic pathways have been identified in blood samples that can be used in the early diagnosis of breast cancer. Of the six metabolites, ethyl (R)-3-hydroxyhexanoate, caprylic acid, and hypoxanthine are noteworthy. Of the eight metabolic pathways identified, fatty acid and aminoacyl-tRNA biosynthesis and inositol phosphate metabolism are the pathways most implicated in the early diagnosis of BC (90). Alterations in lipid metabolism favor processes such as growth, proliferation, and motility of cancer cells, favoring tumor progression (91). The detection of lipids in human plasma samples has led to the identification of diagnostic biomarkers that reflect the early stage of TNBC (ES-TNBC). Diacylglycerol (DG) 34:2 is significantly downregulated in the TNBC subtype. Furthermore, a panel of 5 lipids (DG 34:2, PC 40:3, PC 39:8, PC 34:0 and PC 38:9) can differentiate TNBC from non-TNBC and ES-TNBC from ES-non-TNBC. Finally, in TNBC, ceramides are upregulated, whereas DG and LysoPC are downregulated and PC fluctuates (92) (**Figure 2A**).

## 4 Omics data on the progression of breast cancer

Genomic evidence suggests that IDC is a consequence of DCIS progression. Genes and/or signaling pathways are altered during tumor progression. The most significant changes occur during the transition from normal tissue to carcinoma *in situ*. In addition to the loss of *LIF*, *IL-6*, *HIN-1*, *IL-8*, *GROα*, *GROβ* and *MIP3α* in carcinomas, glutamine synthase (*GS*) and desmoplakin (*DSP*) are the only two genes specifically upregulated in DCIS, while the metabolic enzymes 3-phosphoglycerate dehydrogenase and glyceraldehyde dehydrogenase and mitochondrial NADH: Ubiquinone dehydrogenase and NADH dehydrogenase 1α, have been observed in invasive carcinomas (85). Videlicet, cancer cells have important metabolic alterations (**Figure 2B**).

Downregulated genes in DCIS include *TM4SF1*, *NFKB1A*, *PBEF*, *RASD1*, *TNFRSF10B*, *TNFAIP*, *CLU*, *NSEP1*, LITAF/PIG7, *BZW1*, and *CCNL1*, as well as genes encoding several cytokines and chemokines such as *ILF2*, *IL13RA1*, *LIF*, *CLC*, *CCL2*, and *CXCL1*. Some transcripts are frequently overexpressed in the DCIS such as *PKD1-like*, *STARD10*, *EPS8L2*, and *KIAA0545*. Some of these genes are associated with nuclear factor kappa-light-chain-enhancer of activated B cells (NFκB) and tumor necrosis factor (TNF) pathways, resulting in impaired apoptosis and sustained proliferation of breast cancer cells (93). In IDC, upregulated genes can be grouped into genes related to cell cycle, extracellular matrix or secreted proteins, cell adhesion and motility, and signal transduction. Several underexpressed genes have also been detected in IDC, such as *TM4SF1*, *TRAF4*, *PPARBP*, *AKR1A1*, *RSRP1*, *MAP1LC3A* and *RBBP6* (93). As for ILCs, they are characterized by *CDH1* alterations, as well as dysregulation in the PI3K/Akt pathway due to mutations in *PIK3CA*, *PTEN* alterations, and mutations in *AKT1* (94) (**Figure 2B**).

Transcriptomic analysis of tumor cells and their corresponding adjacent microenvironment cells [belonging to the Molecular Taxonomy of Breast Cancer International Consortium “METABRIC” cohort (95)] have shown that *CDH1* mutation may deregulate immune cells in the tumor microenvironment (96). Expression of genes encoding the α and β subunits of the integrins *ITGA4*, *ITGB2*, *ITGAX*, *ITGB7*, *ITGAM*, *ITGAL* and *ITGA8* correlates positively with the presence of immune cell infiltrates in the tumor, with markers of T cell activation and antigen presentation, and with immunosurveillance gene signatures. Expression of these integrins indicates a favorable prognosis in TNBC and HER2-enriched breast cancers. In contrast, expression of *IBSP*, *ITGB3BP*, *ITGB6*, *ITGB*1 and *ITGAV* predict a poor outcome (97) (**Figure 2B**).

Epigenetic modifications also contribute to breast cancer progression. For example, DNA methylation causes transcriptional silencing of tumor suppressor genes such as *RASSF1A*, *RARB*, *SFN* and *TGM2* (98). Genome-wide methylation analysis revealed that CpG sites were hypermethylated and hypomethylated after *CRY2* silencing. These data suggest that the absence of *CRY2* causes epigenetic dysregulation of genes leading to breast cancer progression (99). The differences in miRNA expression profile are greater in IDC than in ILC compared to their respective carcinomas *in situ*. Thus, IDC is characterized by upregulation of let-7d, miR-210 and miR-221 and downregulation of miR-10b, miR-126, miR-143, miR-218, and miR-335-5p. In contrast, ILC is characterized by upregulation of miR-9, miR-375, miR-182 and miR-183 (57) (**Figure 2B**).

Proteomic analysis of plasma samples from hereditary BC patients carrying a mutation in the *BRCA1* gene has shown that gelsolin, whose loss negatively correlates with tumor progression, is downregulated in these samples. In addition, its levels are associated with *BRCA1* mutational status (100) (**Figure 2B**). Furthermore, *O*-GlcNAcylation is increased in primary malignant breast tumors, and this increase is associated with increased expression of *O*-GlcNAc transferase in grades II and III breast tumors (31).

Metabolomic and transcriptomic data integration studies have enabled the identification of genes, pathways, and metabolites as a part of a cancer prediction model and a better understanding of cancer progression. For example, adenosine monophosphate deaminase 1 (*AMPD1*) and ribonucleotide reductase regulatory subunit M2 (*RRM2*), which are involved in purine metabolism, have been associated with survival in breast cancer patients. Therefore, dysregulation of the purine metabolism pathway may influence breast cancer progression (101). Lipidomic analysis has identified two biomarkers capable of differentiating benign from malignant breast tumors: phosphatidylinositol (PI) (16:0/16:1) and PI (18:0/20:4). In addition, PI (16:0/18:1), phosphatidylglycerol (36:3) and glucosylceramide (d18:1/15:1) have been identified as potential biomarkers for assessing the degree of malignancy of breast tumors (102) (**Figure 2B**).

Microbiota dysbiosis contributes to breast cancer progression through its effects on skin and breast tissue. The presence of *Listeria fleischmannii* in breast tumor tissue is associated with epithelial-mesenchymal transition (EMT), whereas *Haemophilus influenza* correlates with tumor growth, cell cycle progression, E2F signaling, and mitotic spindle assembly (51). Alterations in the gut microbiome alter the production of bacterial metabolites that may influence tumor progression in breast cancer. Uric acid, glycolic acid, d-mannitol, 2,3-butanediol and trans-ferulic acid exert cytostatic effects, while 3-hydroxyphenylacetic acid, 4-hydroxybenzoic acid and vanillic acid stimulate breast cancer proliferation *in vitro*. In addition, 3-hydroxyphenylacetic acid, 4-hydroxybenzoic acid, 2,3-butanediol and hydrocinnamic acid inhibit EMT, and 2,3-butanediol has both cytostatic and anti-EMT properties (103). Gut microbiota may affect tumor progression by influencing the cancer-immunity dialogue. Gut bacteria elicit a complex and coordinated set of innate and adaptive immune responses to maintain tissue homeostasis. Consequently, when the microbiota-host balance is disrupted and dysbiosis occurs, increased production of inflammatory mediators is observed, which is associated with cancer progression. Low diversity in the gut microbiome is associated with decreased lymphocyte level and increased number of neutrophils, as well as decreased survival in breast cancer patients (51) (**Figure 2B**).

## 5 Omics data in relapsed and/or metastatic breast cancer

Between 7% and 11% of early breast cancers recur locally within 5 years after treatment, and 20% to 30% of primary breast cancers develop distant metastases. According to a study based on Surveillance, Epidemiology, and End Results Program (SEER) data for 2010-2013, the most common sites of breast cancer metastasis are bone (30-60%), lung (21-32%), liver (15-32%), and brain (4-10%) (104). Different molecular subtypes of breast cancer are associated with organ-specific metastases. Thus, luminal subtypes A and B metastasize primarily to the bone, the HER2-enriched subtype to the brain and liver, and TNBC to the lung (105). Genomic analyses have shown that metastases retain the same molecular subtype and prognostic signature as their primary tumors. These data suggest that metastatic potential is already determined in the primary tumor. The poor prognostic signature consists of genes that regulate cell cycle, invasion, metastasis, and angiogenesis (106, 107). Common alterations in *TP53* (51%) and *PIK3CA* (49%), as well as mutations/deletions in *NF1* (15%), mutations in *PTEN* (10%), and mutations/deletions in *ARID1A* (15%) have been identified in metastatic breast cancer tumors (108). Bone metastases of breast cancer are characterized by increased expression of the *TFF1*, *TFF3*, *AGR2*, *NAT1* and *CR1P1* genes, as well as the chemokine receptors CXCR4 and C-C chemokine receptor type 7 (CCR7), and upregulation of the zinc finger protein SNAI1 (SNAI1) (45, 109), which is involved in the induction of EMT (110). In breast to lung cancer metastasis, a number of genes such as *MMP1*, *MMP2*, *CXCL1*, *PTGS2*, *ID1*, *VCAM1*, *EREG*, *SPARC*, and *IL13RA2* have been identified in breast to lung cancer metastasis that promote and are clinically correlated; as well as the mitogen-activated protein kinases (MAPK), NFκB and vascular endothelial growth factor (VEGF) signaling pathways (111) (45). Mutations in the *ESR1*, *AKT1*, *ERBB2*, and *FGFR4* genes have been observed in metastatic breast tumors in the liver (45, 112, 113). Transcriptome analysis revealed that the TNF-α pathway is upregulated in lung metastases compared to liver metastases (114). *ATAD2*, *DERL1* and *NEK2A* have been shown to be overexpressed (115–117), whereas *ATM*, *CRYAB* and *HSPB2* genes are often suppressed and/or underexpressed in breast cancer metastases to the brain (45, 118, 119), (**Figure 2C**). The AURORA study, consisting of genomic and transcriptomic profiling in matched primary tumors and early metastases, has described the key role of somatic mutations *GATA1* and *MEN1* in metastasis. In addition, the enrichment of *ESR1*, *PTEN*, and *PIK3CA* in metastases have been determined, as well as *CDH1* and *RB1* mutations, *MDM4* and *MYC* amplifications, and *ARID1A* deletions (120). Of note, *TP53* mutations and *MYC* amplification are associated with shorter time to relapse (25). Eight common genes have been found to have significant effects on TNBC survival (*ELOB, SLC39A7, TIMM13, BANF1, NDUFS1, NDUFB7, TRAPPC5*, and *MVD*). Finally, a signature of 87 immune genes has been established that is highly predictive of pathologic complete response (pCR), which in turn correlates with improved OS and distant metastasis-free survival (DMFS) (121). These 87 immune genes are shown in **Figure 2C**.

The immune system is involved in the development of cancer, from tumor initiation to metastasis (122). Patients with stage 3 and 4 breast cancer have a higher percentage of immunosuppressive cells (granulocytic myeloid-derived suppressor cells (MDSCs), CD14^+^CD16^+^ intermediate monocytes, and CD127^−^CD25^high^FoxP3^+^ Treg cells). Inflammation-related genes are differentially expressed in TNBC. In fact, low expression of CD163 and CXCR4 together with high expression of thrombospondin 1 (THBS1) correlates with an increased risk of relapse and poor survival in TNBC (123). The proinflammatory cytokines serum amyloid A (SAA) and IL-18 are elevated in the serum of patients with recurrent breast cancer. Therefore, SAA and IL-18 may be prognostic markers for breast cancer recurrence (124) (**Figure 2C**).

miRNAs are also involved in cancer migration and metastasis. miR-21, miR-10b, miR-373 and miR-520c promote metastasis, whereas miR-126, miR-335, miR-31, miR-146a, and miR-497 suppress metastasis. miR-9 is associated with local recurrence and ER+ tumors, whereas miR-10 is involved in cell proliferation, migration, and invasion (57). On the other hand, *Cyclin D2*, *RAR-beta*, *Twist*, *RASSF1A* and *HIN-1* genes show increased methylation in bone, brain and lung metastases compared to primary breast cancer, with *HIN-1* and *RAR-beta* methylation significantly higher in each group (125). Hypermethylation and downregulation of genes involved in breast cancer progression or EMT, such as *LYN, MMP7, KLK10* and *WNT6*, are associated with a significantly lower risk of metastatic relapse (126). Furthermore, H3K4 acetylation has been correlated with breast cancer progression, estrogen responsiveness, and the oncogenic EMT pathway. Therefore, H3K4 is a potential biomarker for tumor progression leading to aggressive metastatic phenotypes (127) (**Figure 2C**).

Other biomarkers have been identified using other omics techniques. As mentioned above, E-cadherin is considered a good prognostic marker in non-invasive breast cancer, and loss of E-cadherin protein is one of the main features of EMT (82). Furthermore, a metabolomic study has identified 9-cis-retinoic acid as a critical metabolite in breast cancer progression, as it significantly decreased during breast cancer progression to metastasis. This suggests that 9-cis-retinoic acid inhibits tumor progression to metastasis, probably by attenuating cell invasion and migration (128). Combining lipidomic techniques with transcriptomic analysis, PI (18:0/20:3) accumulation has been found to be associated with an increased incidence of lymph node metastasis and activation of the PD-1-related immune checkpoint pathway (129).

Finally, alterations in the microbiota have also been observed to influence breast cancer metastasis. The presence of *Bacteroides fragilis*, a gut-colonizing bacterium, can induce epithelial hyperplasia to promote tumor growth and metastasis via the β-catenin–Notch1 axis (130). In addition, *Fusobacterium nucleatum* promotes tumor progression and metastasis (131) (**Figure 2C**).

## 6 Omics and treatment implications

Molecular changes that occur during cancer treatment determine the response to different therapies and guide the optimal choice of treatments to reduce local recurrence and distant metastasis, thereby increasing disease-free survival. The various omics are key to identifying these molecular changes. In fact, several clinical trials are based on one or more omics technologies.

Classically, aromatase inhibitors are recommended for patients with ER+ metastatic breast cancer because they suppress estrogen production. Trastuzumab and lapatinib are administered to patients with HER2-enriched breast tumors, as trastuzumab is a humanized monoclonal antibody against the extracellular domain of HER2, and lapatinib is a tyrosine kinase inhibitor that blocks both HER2 and EGFR activation. However, in light of the omics findings, more refined treatments may be considered according to genomic alterations. For example, both luminal subtypes frequently harbor mutations in *PIK3CA*, so inhibitors of this kinase may be a therapeutic target; *MYC* amplification suggests the use of platinum analogs and taxanes; and, patients with *BRCA1/2* mutations may benefit from poly ADP ribose polymerase (PARP) inhibitors and/or platinum compounds (44). The RAS/RAF/MEK/ERK signaling pathway is altered in tumors that overexpress EGFR and HER2. Since this kinase cascade is critical for survival and apoptosis, alterations in this pathway could affect sensitivity/resistance to anticancer therapies. Mutations in *KRAS*, *HRAS*, *NRAS*, *BRAF* and *NF1* have been observed in all breast cancer subtypes, although their frequency does not exceed 4%. However, copy number alterations (CNAs) of *KRAS* and *BRAF* have been observed in TNBC. Therefore, the use of inhibitors of these kinases in combination with other therapies may delay or induce resistance to treatment (132). *ESR1* methylation is considered a good predictor of survival in tamoxifen-treated patients, whereas *ARHI* methylation predicts survival in non-tamoxifen-treated patients. *BRCA1* hypermethylation, in turn, sensitizes TNBC patients to DNA-damaging chemotherapeutic agents such as cisplatin and PARP inhibitors (77). The prevalence of the PI3K/AKT/mTOR signaling axis has been observed in TNBC. The c-Kit protein, a receptor tyrosine kinase involved in the initiation of this cascade, is overexpressed in 20-25% of TNBC, hence, the tyrosine kinase inhibitors (TKIs) such as imatinib or sunitinib are used in the treatment of these cancers (133) (**Table 4**).

**Table 4:** Refined treatments that take into account genomic studies.

Alteration	Treatment	REF
Mutations in PIK3CA	Inhibitors of PI3KA	(44)
Myc amplification	Platinum analogs and taxanes	(44)
Mutation in BRCA1/2	PARP inhibitors and platinum compounds	(44)
BRCA1 hypermethylation	Cisplatin, PARP inhibitors	(77)
c-Kit overexpression	Imatinib, sunitinib	(133)

The endoplasmic reticulum protein KIAA1199, which is involved in tumor growth and invasiveness, is significantly overexpressed in breast tumor samples. Therefore, KIAA1199 is considered a new target for biomarker development and a novel therapeutic target for breast cancer (134, 135). Similarly, cofilin-1 (CFL-1), interleukin-32 (IL-32), proliferating cell nuclear antigen (PCNA), syntenin-1 (SDCBP), and riboforin-2 (RPN-2) have been identified as potential target antigens for HLA-A2+ TNBC immunotherapy (136), or the proto-oncogene RET and kallikrein (KLK)8 as antigens associated with breast cancer in general (137).

**Figure 3:**
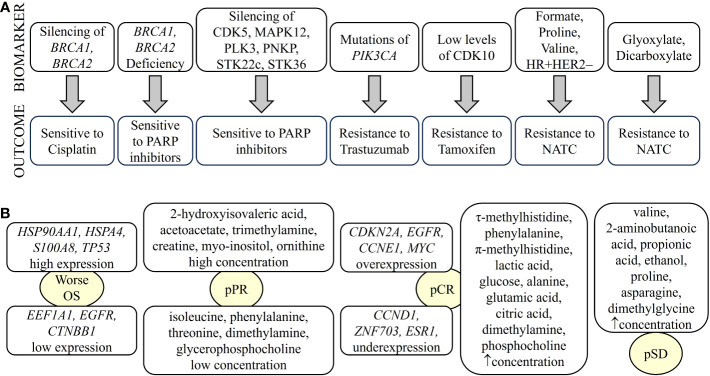
Biomarkers of breast cancer defining treatment outcome detected by omics. Biomarkers identified by different omics can determine drug sensitivity/resistance **(A)** or type of response to treatment **(B)**. NATC, neoadjuvant targeted therapy; pCR, pathological complete response; pPR, pathological partial response; pSD, pathological stable disease. “up arrow” means increase.

Metabolic and lipidomic profiling of TNBC samples combined with transcriptomic and genomic data have identified a number of metabolites as potential therapeutic targets for different transcriptomic subtypes of TNBC. The LAR subtype is characterized by an enrichment of ceramides and fatty acids. Therefore, sphingosine-1-phosphate (S1P), an intermediate of the ceramide pathway, may be a promising drug for the treatment of LAR tumors. In contrast, the basal-like immunosuppressed transcriptomic subtype (BLIS) is characterized by increased metabolites related to oxidation reaction and glycosyl transfer and the lowest level of metabolic dysregulation. In this case, N-acetyl-aspartyl-glutamate has been identified as a key tumor-promoting metabolite and a potential therapeutic target for high-risk BLIS tumors (138). Furthermore, high levels of sphingomyelins are associated with improved DFS in patients with TNBC. Therefore, sphingomyelins and enzymes involved in sphingolipid metabolism could be considered as prognostic markers and potential therapeutic targets, respectively (139). Omics studies can also identify specific pharmacological pathways of resistance and sensitivity of tumor cells to different therapies. For example, a combination of *TP53* deficiency and silencing of *BRCA1*, *BRCA2*, or BRCA1/2-associated genes results in cisplatin sensitivity (140). *PI3KCA* mutations alone or in combination with *PTEN* appear to predict worse outcome after trastuzumab monotherapy or in combination with chemotherapy (141). *BRCA1/2*-deficient cells are sensitive to PARP1 inhibitors. In addition, silencing of kinases such as cyclin-dependent kinase 5 (CDK5), mitogen-activated protein kinase 12 (MAPK12), polo-like kinase 3 (PLK3), polynucleotide phosphatase/bifunctional kinase (PNKP), serine/threonine kinase (STK)22c, and STK36 strongly sensitize to PARP inhibitors (142). Cyclin-dependent kinase (CDK)10 has been identified as a determinant of resistance to endocrine therapies such as tamoxifen, as low CDK10 levels lead to early on tamoxifen treatment (143) (**Figure 3A**). The classic treatment for HR+ breast cancer is endocrine therapy. However, approximately 30% of patients develop resistance to endocrine therapy. A transcriptomic/proteomic study has identified the set of candidate genes *CEACAM1, KRT19, TMEM81, TMEM119, ESRRA, ERBB3, SRC, AKT1S1, SGEF, SCG5, ALOX12B, CKB, BID, XRCC1, NSL1*, and *CHEK2* that are able to discriminate progression/resistance (PD) from complete response (CR) and correlate significantly with survival (144). In addition, 298 differentially expressed genes were identified between drug-sensitive (DS) and drug-resistant (DR) breast cancer patients prior to neoadjuvant treatment. Among them, the peptidyl-prolyl cis-trans isomerase FKBP4 (FKBP4) and the protein S100-A9 (S100A9) could be putative predictive markers to distinguish the DR group from the DS group of breast cancer patients (145).

Metabolic studies have identified biomarkers that predict response to treatment. For example, glycohyocholic and glucodeoxycholic acids can stratify TNBC patients according to response to neoadjuvant chemotherapy and OS (146). On the other hand, when comparing patients with large primary breast cancer who had received neoadjuvant chemotherapy plus bevacizumab with those who had received chemotherapy alone, higher levels of leucine, acetoacetate and trihydroxybutyrate and lower levels of formate were observed 12 weeks after treatment (147). Furthermore, baseline immunometabolic assessment in combination with ER status could predict the response to neoadjuvant targeted chemotherapy (NATC) based on patient trastuzumab-paclitaxel combination and disease relapse in HER2+ patients. HER2+/ER+ patients have higher levels of T-cell stimulating factors, but also higher levels of cytokines that might be responsible for T-cell suppression. The combination of metabolic data with IL-2 and IL-10 cytokine levels has been shown to be prognostic for relapse (148). In addition, serum metabolites such as leucine, formate, valine, and proline, along with hormone receptor status, have been shown to be discriminators of NATC response. For example, formate, proline, valine, HR+, and HER2− are directly associated with NATC resistance. In contrast, leucine, HR− and HER2+ are directly related to NATC sensitivity. In addition, glyoxylate and dicarboxylate metabolism have been implicated in NATC resistance (149) (**Figure 3A**).

Pathologic complete response (pCR) and residual disease have been correlated with the genome, transcriptome, and tumor immune microenvironment in patients with early and locally advanced breast cancer undergoing neoadjuvant therapy (150). The pCR is associated with overexpression of driver genes such as *CDKN2A*, *EGFR*, *CCNE1*, and *MYC* and underexpression of *CCND1*, *ZNF703*, and *ESR1*, as well as increased immune activation characterized by enrichment of innate and adaptive immune cells. However, tumors with residual disease, particularly HER2− tumors, showed enrichment of EMT and attenuated immune response due to the enrichment of inhibitory CD56^dim^ natural killer cells and regulatory T cells, leading to therapy resistance (150). In transcriptomic profiling, *HSP90AA1*, *EEF1A1*, *APP* and *HSPA4* were associated with recurrence in breast cancer patients with pCR due to neoadjuvant chemotherapy. *TP53*, *EGFR*, *CTNNB1*, *ERBB2* and *HSPB1* may play an important role in the survival of pCR patients. Patients with tumors expressing high levels of *HSP90AA1*, *HSPA4*, *S100A8*, and *TP53* and low levels of *EEF1A1*, *EGFR* and *CTNNB1* showed significantly worse overall survival (OS) (151) (**Figure 3B**). A set of genes including *AKT1S1*, *NSL1*, *ESRRA*, *TMEM81*, *CKB*, *SGEF*, *KRT19*, *SCG5*, *CEACAM1*, *ALOX12B*, *IDB*, *SRC*, *CHEK2*, *ERBB3* and *XRCC1* have been identified as critical targets of both selective estrogen receptor modulators (SERMs)/selective estrogen receptor downregulators (SERDs) and aromatase inhibitors (AIs) of endocrine resistance (144).

A systemic metabolic study showed that patients with large primary breast cancers undergoing neoadjuvant chemotherapy with poor response have higher citrate levels and lower histidine levels (147). In addition, TNBC patients with pCR had elevated levels of circulating τ-methylhistidine, phenylalanine, π-methylhistidine, lactic acid, glucose, alanine, glutamic acid, citric acid, dimethylamine and phosphocholine, whereas patients with or pathological stable disease (pSD) had elevated levels of valine, 2-aminobutanoic acid, propionic acid, ethanol, proline, asparagine, and N,N-dimethylglycine. Finally, TNBC patients with pathologic partial response (pPR) had high levels of 2-hydroxyisovaleric acid, acetoacetate, trimethylamine, creatine, myo-inositol and ornithine, but low levels of five metabolites, namely isoleucine, phenylalanine, threonine, dimethylamine and glycerophosphocholine (**Figure 3B**). Therefore, we can infer that alterations in the pathways of glycine, serine, and threonine metabolism; valine, leucine, and isoleucine biosynthesis; and alanine, aspartate, and glutamate metabolism could be used as potential models to predict whether a patient with TNBC is suitable to receive neoadjuvant chemotherapy (152).

The relevance of all these biomarkers lies in their translational potential to identify specific treatments for breast cancer. Several clinical trials are ongoing. A Phase I program conducted at the University of Texas MD Anderson Cancer Center demonstrated that patients who received alteration-matched therapy had a higher objective response rate (ORR), PFS, and OS compared to unmatched therapy (153).

One of the most important clinical trials based on genomic studies is the “Microarray In Node Negative Disease may Avoid ChemoTherapy” (MINDACT). The MINDACT clinical trial aims to demonstrate the clinical relevance of the 70-gene prognostic signature (or MammaPrint™) determined by van’t Veer (106) and to compare it with the traditional clinicopathologic prognostic indicators for the assignment of adjuvant chemotherapy in patients with node-negative breast cancer (154). As a result, MammaPrint™ was found to be effective as traditional tools in identifying high-risk patients, but more accurate in identifying low-risk patients who could avoid adjuvant chemotherapy (155, 156). This gene signature outperforms all traditional clinical prognostic factors and clearly discriminates patients with an excellent prognosis from those at high risk of recurrence (157, 158). Phase III results showed that approximately 46% of women with clinically high-risk breast cancer are unlikely to need chemotherapy (159). Women with high clinical risk and low genomic risk younger than 50 years had excellent DMFS when treated with endocrine therapy alone (160). In addition, patients with ER+, HER2− and stage I lymph node-negative tumors ≤2 cm treated with endocrine therapy had significantly fewer breast cancer events (161). On the other hand, no improvement in outcomes was observed with the use of docetaxel-capecitabine compared with anthracycline-based chemotherapy (162). Last year (2022), the Austrian Group Medical Tumor Therapy prospective registry confirmed that the addition of MammaPrint™ to the routine treatment of early luminal breast cancer yields clinically useful results (163).

Concurrent with the MINDACT clinical trial in Europe, the Trial Assigning Individualized Treatment Options Rx (TAILORx) was conducted in North America with the goal of reducing chemotherapy overtreatment by integrating molecular diagnostic testing into the clinical decision-making process. TAILORx is based on a 21-gene based assay (Oncotype DX™) that calculates a recurrence score (RS) and reserves chemotherapy for patients with a low RS (164). Women with a high RS who were treated with adjuvant chemotherapy regimens containing taxanes and/or anthracyclines plus endocrine therapy had an estimated 5-year freedom rate from distant breast cancer recurrence of 93% (165). In patients with ER+, HER2−, lymph node-negative and intermediate RS, adjuvant endocrine and chemoendocrine therapy had similar efficacy, although chemotherapy had some benefit in some women aged 50 years or younger (166). Among women with intermediate RS, Hispanic ethnicity and Asian race were associated with better outcomes. However, Black race was associated with worse clinical outcomes and did not benefit from adjuvant chemotherapy (167).

Other studies are the aforementioned AURORA US Metastatasis project and the TransNEO study. The AURORA US Metastasis project conducted a multiomic study including genomics, epigenomics and transcriptomics in primary tumors and their corresponding metastatic breast cancers (168). In metastatic TNBC, significantly lower expression of MHC class I genes (HLA-A, HLA-B, and HLA-C), DNA methylation of HLA-A, and small focal HLA-A were observed, which were associated with lower immunity and worse OS. Tumors with DNA-methylated HLA-A could be targeted for DNA demethylating drugs in combination with immune checkpoint inhibitors (ICI) (168, 169). The TransNEO molecular profiling study of patients with early and locally advanced breast cancer undergoing neoadjuvant therapy. Genomic, transcriptomic, and tumor immune microenvironment data were combined with clinical and digital pathology data to perform machine learning to create a predictive pCR model. This model is robust and could guide treatment selection in future clinical trials, including in the context of adjuvant therapy (150).

## 7 Conclusion and perspectives

Currently, treatment selection in breast cancer patients is based on broad clinicopathologic features that fail to accurately classify patients into risk groups, hence resulting into overtreatment of vast segments of patients. Data suggest that ORR, DFS and OS can be improved by the use of tailored therapies. Improved outcomes depend on the identification of new biomarkers that allow for the stratification of patients and eligibility for new therapies. Besides, the identification of biomarkers that predict treatment efficacy will minimize side effects or cumulative toxicity in patients unlikely to benefit from such treatments. Clinical trials, which thus far have mostly been based on genomic, transcriptomic and/or proteomic studies, have been effective in assigning treatment. It is our understanding that multiomics studies, including other omics techniques such as metabolomics, immunomics or microbiomics, are an important step towards precision medicine and hence refine the assignment of the best possible treatment for each patient. Special attention should be paid to the statistical methods used in the analysis of multiomic data to avoid spurious correlations. In fact, correlation coefficients should not be used to explain a process, such as cancer progression, in which multiple variables are involved. In these cases, the use of regression or multivariate analysis techniques will be more appropriate. In addition, validation cohorts will be needed to confirm the reproducibility, robustness, and validity of the results. It is important that validation cohorts have a pre-calculated sample size using statistical power tests and that minimum assay quality criteria have been established. In conclusion, we anticipate that clinical trials based on high-dimensional multiomics data interpreted by artificial intelligence will guide each patient to an optimized and personalized treatment, that will avoid overtreatment, minimize side effects, and improve both DFS and OS.

## Author contributions

LA: Conceptualization, Investigation, Writing – original draft, Writing – review & editing. DB: Validation, Writing – original draft, Writing – review & editing. MD: Validation, Writing – original draft, Writing – review & editing. MI: Validation, Writing – original draft, Writing – review & editing. GK: Validation, Writing – original draft, Writing – review & editing. RP: Validation, Writing – original draft, Writing – review & editing. LS: Conceptualization, Funding acquisition, Investigation, Resources, Supervision, Writing – original draft, Writing – review & editing.
